# Comparison study of QuantiFERON test with tuberculin skin testing to diagnose latent tuberculosis infection among nurses working in teaching hospitals of Ahvaz, Iran.

**Published:** 2016

**Authors:** Shokrollah Salmanzadeh, Hajar Abbasissifar, Seyed Mohmmad Alavi

**Affiliations:** 1 Health Research Institute, Infectious and Tropical Disease Research Center, Joundishapur University of Medical Sciences, Ahvaz, Iran.; 2 Joundishapur University of Medical Sciences, Ahvaz, Iran.

**Keywords:** Latent tuberculosisinfection, Quanti FERON-TB test, Tuberculin skin test, Health care workers

## Abstract

**Background:**

Prompt diagnosis and treatment of latent tuberculosis (TB) infection (LTBI) are needed to control TB. The aim of the study was to compare the performance of Quanti FERON-TB test (QFT) with conventional TST for the diagnosis of LTBI.

**Methods:**

In this analytical - comparison study, we enrolled 87 nurses working in teaching hospitals in Ahvaz. All study subjects were tested by TST. TST results were interpreted as positive if induration was more than 10 mm. If the level of QFT after stimulation was equal or greater than 0.35 IU/ml, test was considered as positive. Data were analyzed with SPSS program. QFT results compared with induration in TST and its relation to all variables were investigated.

**Results:**

The rate of LTB diagnosis by TST and QFT was 31% and 35.6%, respectively. There was no significant difference between TST and QFT in LTB diagnosis (P=0.62). Among the 56 subjects who were TST- negative, 14 cases (approximately 25%) were QFT- positive and 42 (75%) were QFT- negative. Among the 31 cases (35.6%) that had TST- positive, 13 (42%) were QFT-positive and 18 (58%) were QFT- negative. The overall percent agreement was 63.2% (k=0.139, P=0.69), discordance %=15.9-20.7, sensitivity= 41.5% and specificity=75.5%.

**Conclusion:**

Diagnostic value of QFT is similar to TST, when there is strong clinical and epidemiological evidence of LTB in a nurse with negative TST, adding QFT to diagnostic evaluation is associated with increased rate of LTB diagnosis.

Tuberculosis (TB) is a major public health problem throughout the world. TB remains as an important infectious disease which causes a high morbidity and mortality worldwide (1). World Health Organization (WHO) estimates that one-third of the world’s population is infected by M*. tuberculosis *with an annual incidence of 8 –9 million new TB cases (2). TB continues to be a major infectious disease in Iran despite the implementation of a national tuberculosis program (NTP) (3). Health care workers (HCWs) because of occupational exposure to tuberculosis patients are at an increased risk for *M. tuberculosis* infection. HCWs particularly nurses working in infectious disease ward, pulmonary department, medical intensive care unit and emergency room have been reported to be at a higher risk of developing TB than other HCWs working in other parts of hospital due to increased possibility of TB transmission (4).

Latent TB infection (LTBI) diagnosis in nurses is crucial because of the risk of progression to active TB, so prompt diagnosis and treatment of LTBI are needed to TB control (5-7). The tuberculin skin test (TST) is still the main diagnostic tool for the detection of LTBI, although it is associated with some limitations of false positive and false negative results (8, 9). In recent years, several novel diagnostic testssuch as interferon-release assays (IGRAs), Quanti FERON-TB Gold assay (QFT) have been suggested for LTBI (1, 2, 4, 10-12).

Several systematic reviews have suggested that QFT is as sensitive as and more specific than the TST in LTBI diagnosis. Search in medical publications for screening TB among high risk population by QFT shows a gap in the existing literature. However, the use of IGRAs for routine screening of HCWs remains an area of controversy (1,2).Since, there are limited data describing alternative diagnostic tools for LTBI for TB-risk associated individuals in Khuzestan, the present study was conducted to compare performance of Quanti FERON-TB test (QFT) with conventional TST for the diagnosis of LTBI.

## Methods

In this analytical - comparison study, the study population was all hospital nurses who had not an apparent underlying disease and did not receive immunosuppressive treatment during the study. Of the 98 nurses who wished to participate in the study, 11 cases were excluded due to underlying disease (3 bronchial asthma, 6 rheumatoid arthritis and 2 inflammatory bowel disease) or were receiving corticosteroids. Finally we enrolled 87 nurses working in teaching hospitals in Ahvaz. All subjects had negative serological findings for human immunodeficiency virus (HIV) and absence of obvious risk factors for that disease. Data regarding demographic; radiologic and clinical information were collected for all the participants. After obtaining informed consent from each participant, all study subjects were tested by TST using 0.1 mill of 50 units’ solution of tuberculin salute by Mantua’s method (intradermal injection). After 48 to 72 hours, the largest diameter of skin induration was measured in millimeters. The results of this test were interpreted using the manufacture's brochure and NTP. TST results were interpreted as positive if induration was more than 10mm. After 48 hours of TST, QFT testing for all patients was performed according to the manufacturer's instructions. This test consists of a text control (Panel: peripheral blood without antigens or mitogen) A ratio control (mitogen-stimulated peripheral) Wake-stimulated peripheral blood antigens of mycobacterial ESAT-6 AndCPF-10.

Peripheral blood samples obtained from each subject were incubated for 20 hours at 37 °, INF-γ levels in the sample panel was considered as background. If the level of INF-γ after stimulation was equal or greater than 0.35 IU/ml, test was considered as positive. If the level of INF-γ was less than 0.35 IU/ml it was considered as negative. And if the antigen-stimulated sample was negative and the positive control test was less than 0.5 IU/ml, test was considered as indeterminate. Ultimately, indeterminate results were excluded and only the positive and negative results include the statistical analysis. Then results of experiments and clinical history were recorded.

Latent tuberculosis was confirmed if the patient was infected with *M.tuberculosis* but had no signs and symptoms or radiological findings consistent with active pulmonary or extrapulmonary TB. Cases with at least two sputum smear positive for acid fast bacillus (SSP-AFB) or a chest radiography suggestive of tuberculosis plus one SSP-AFB were considered as pulmonary TB. Confirmed pulmonary or extrapulmonary TB was based on sputum or tissue culture positive for *M. tuberculosis.*

Data were analyzed in SPSS 16. A chi-square test was used to compare proportions, differences with p value less than 0.05 were considered statistically significant. Concordance between TST and QFT results was evaluated using agreement and kappa statistics. QFT results compared with induration in TST and its relation to all variables are investigated.

## Results

Of the total 87 studied nurses, 66 (76%) of these were females and 21 (24%) were males. The range of age was between 24 to 49 years old with mean (mean±SD) age of 28.6±10.5 years old.14 subjects (16.1%) had at least one of the symptoms of fever, cough, sputum or night sweats, and 73 (83.9%) did not have any of the above symptoms.After 48 h of intradermal injection of soluble PPD (TST) in 32 subjects (38.6%), induration diameter was less than 5 ml, 24 (27.5%) between 5 to 10 mm, 11 (12.6%) between 10 to 15 mm and ultimately 20 (23.1%) above 15 mm. Of the total 87 subjects, 26 subjects (30%) had positive QFT result and 61 (70%) had negative results. according to the CDC instructions for the subjects, (hospital staff) TST above 10 mm was considered positive. Therefore, according to cut-off value of 10 mm QFT results were compared with TST. The rate of LTB diagnosis by TST and QFT was 31% and 35.6%, respectively. There was no significant difference between TST and QFT in LTB diagnosis (P=0.62). 

Among the 56 subjects who were TST- negative, 14 cases (approximately 25%) were QFT- positive and 42 (75%) were QFT- negative. Among the 31 cases (35.6%) that had TST- positive, 13 (42%) were QFT-positive and 18 (58%) were QFT- negative (table 1). Among the studied cases, we diagnosed tuberculosis infection with different results by the two tests, 14 cases were QFT positive but TST negative and 18 cases were QFT negative but TST positive.

Clinical findings of weight loss, cough or night sweats were observed in 2 (14.3%) QFT-positive subjects and in 12 QFT - negative ones. Of the 2 cases that had positive QFT and clinical symptoms, 1 individual (50%) was TST- positive and 1(50%) was TST- negative. Of the 12 cases with clinical symptoms and negative QFT, 9 (75%) were TST- negative and 3 (25%) were TST-positive (table 2). Of the total 87 cases, 73 (83.9%) were asymptomatic, among them, 24 (32.9%) were QFT - positive that 11(11.24, 45.8%) of them were TST- positive and 13 (13.24, 54.2%) were TST- negative. Among the 73 patients who had no symptoms, 49 (49.73, %67.2) had negative QFT, among them, 15 (15.49, 30.6%) were TST- positive and 34 (34.49, 69.4%) were TST- negative (table 3). There was no significant difference in agreement between symptomatic and asymptomatic subjects. There was also no significant difference in the agreement between males (64.5%, k=0.20, P=0.45) and females (63.5%, k=0.34, P=1.0). Among the total 87 studied cases, 5 cases were both symptomatic and had positive TB infection tests (2 QFT and 3TST). Among the symptomatic positive cases, only two cases were diagnosed as active pulmonary TB based on positive sputum AFB and culture examination. Pulmonary TB cases were referred to Ahvaz Health Center for treatment.

Of the total cases, 34 (33.1%) of the subjects were in the age group of 21-30 years, 43 (49.4%) age group of 31-40 years and 10 (11.5%) age group of 41-50 years. The results of TST and QFT among the age groups are shown in table 3. There was no significant difference in agreement between subjects in different age groups (P=0.78).

**Table 1:** Agreement between tuberculin skin test (TST) and quantiFERON test (QFT) in diagnosis of LTB

**Test**	**TST positive**	**TST negative**	**Total**	
QFT positive	13 (14.9)	14 (16.1)	27 (31.0)	Agreement %=63.2% (k=0.139,p=0.69) Discordance %=15.9-20.7 Sensitivity=41.5% Specificity=75.5%
QFT negative	18 (20.7)	42 (48.3)	60 (69.0)	
Total	31(35.6)	56(64.4)	87 (100)	

**Table 2:** The results of tuberculin skin test and quantiFERON test in the presence or absence of clinical findings

**Clinical finding**		**TST-positive**	**TST-negative**	**Total**	**Agreement %**
yes	QFT- positive	1 (7.1)	1 (7.1)	2 (14.2)	71.4% (k=0.21,p=1)
	QFT-negative	3 (21.4)	9 (64.4)	12 (85.8)	
	Total	4 (28.5)	10 (71.5)	14 (100)	
No	QFT- positive	11 (15.1)	13 (17.8)	24 (32.9)	61.4% (k=0.21,p=0.83)
	QFT-negative	15 (20.5)	34 (46.6)	49 (67.1)	
	total	26 (35.6)	47 (64.4)	73 (100)	

Abbreviation: K; Kappa coefficient, P; p-value, TST; tuberculin skin test, QFT; quantiFERON test

**Table 3:** The results of tuberculin test and quantiFERON test among the nurse participants studied nurse in different age groups

**Age groups (years)**		**TST- positive**	**TST- negative**	**Total**	**Agreement %**
21-30	QFT- positive	3 (8.8)	6 (17.6)	9 (26.4)	64.7% (k=0.21,p=.078)
	QFT-negative	6 (17.6)	19 (56.0)	25 (73.6)	
	Total	9 (26.4)	25 (73.6)	34 (100)	
31-40	QFT -positive	7 (16.3)	7 (16.3)	14 (32.6)	65.1% (k=0.21,p=0.83)
	QFT-negative	8 (18.6)	21 (48.8)	29 (67.4)	
	Total	15 (34.9)	28 (65.1)	43 (100)	
41-50	QFT -positive	2 (20)	1 (10)	3 (30)	60% (k=0.21,p=0.81)
	QFT-negative	3(30)	4 (40)	7 (70)	
	Total	5 (50)	5 (50)	10 (100)	

Abbreviation: K; Kappa coefficient, P; p-value, TST; tuberculin skin test, QFT; quanti FERON test

## Discussion

Early detection of TB infection and appropriate treatment of individuals with latent TB to prevent progression to active disease is a main strategy to reduce the incidence of TB. Health care workers, especially nurses are among the individuals at higher risk of TB infection acquisition because of daily contact with infected patients.

TST is widely used as a screening test for the diagnosis of latent form of tuberculosis infection. The high coverage of BCG vaccination in Iranian population has been established since 1983 as routine national immunization program and significantly has reduced the efficacy of TST because of the cross-reactivity of tuberculin with the BCG vaccine (13, 14). QFT test is a laboratory test done on a blood sample that detects secreted INF-γfrom lymphocyte, in response to M. tuberculosis-specific antigens which is also suggested for diagnosing LTB. Previous studies suggested that QFT-2G test is not under the influence of past BCG vaccination (13, 14). In the present study, the detection rate of LTBI by TST was 31% while by QFT, was 35.6%. The prevalence of LTBI in nurses in the region of study with the rate of 31-35.6% is significantly higher than the prevalence in the other parts of the world previously reported (1, 10) but similar to reports from countries with moderate to high prevalence of TB (11, 12, 15). The prevalence of LTBI among HCWs is related to epidemiological status of TB in general population, the risk of contact with active TB, years of experience and occupation in hospital wards where pulmonary TB are managed, preventive methods which are used by hospital and presence or absence of TB risk factors.

In the current study, according to poor agreement in comparison to QFT with TST (k=0.139, P=0.69), we found that QFT is not a preferred diagnostic tool for LTB because of low sensitivity and moderate specificity .Our finding on sensitivity and specificity of QFT is similar with the work of some authors from different countries (13, 15). Our results of concordance and discordance of QFT in comparison to TST are in consistent with some studies (1, 7, 11) but in contrast to other studies conducted in different parts of the world (4, 15). Previous studies suggested that in areas with low prevalence of TB and high BCG vaccination rate, QFT is not a useful diagnostic tool for LTB infection (13, 14). But in areas with high TB prevalence, QFT is a reliable tool for diagnosing LTB infection (4, 12, 15). Indeed, as the only diagnostic test, QFT is as effective as TST (8, 9). In our study, the rate of LTB detection rate of TST and QFT was not influenced by age, gender and clinical symptoms. This finding is in contrast with literature and other reports (2, 11). We believe that TB epidemiological pattern in Iran especially in health care setting and hospitals (young and female nurse population) is an acceptable reason for this difference.Although, the QFT test is technically feasible in performance and is said to be a more accurate method in the diagnosis of LTBI than the TST in developed countries, but QFT due to expensiveness, unavailability, difficulty to do it compared to TST, cannot be considered as a useful diagnostic tool for the diagnosis of latent tuberculosis in normal population. Our finding does not agree with some other reports such as the study of Adewole et al. Jo, et al. and Wei, et al. that suggested that QFT be used to diagnose LTBI (4, 12, 15). The reasons for these differences are not definitely clear but may be based upon some factors such as: study design, sample size, kind of used kit, difference in epidemiological pattern of TB in the various regions and proportion of frequent exposures among studied population.

The current study to calculate the concordant and discordant pairs suggests that if QFT is added to TST, detection rate of LTB will increase from 31% to 51.7% [(31+14)/87). We observed that when TST is negative but with strong suspicion of TB infection in at risk population such as nurses with frequent daily exposure to TB sources, furthermore, the approach of using QFT as diagnostic approach is added. The added value of QFT-GIT in the diagnosis of LTBI has been investigated in other studies with different burden and incidence of TB with controversy results (16, 17). More effects of QFT and 81.8% relative increase in LTBI detection were reported when QFT was added to TST (16). 

In conclusion, according to our findings, diagnostic value of QFT is similar to TST, when there is strong clinical and epidemiological evidence of LTB in a nurse with negative TST, adding QFT to diagnostic evaluation is associated with increased rate of LTB diagnosis. 

## Notes

**Funding: **This study was supported in part by the Infectious and Tropical Research Center of Joundishapur University of Medical Sciences. This paper has been derived from the medical doctoral thesis (no: A/836) of Dr Ms. Hajar Abbasi Far a Medical student.

## Notes

**Authors’ Contribution: **SM Alavi; final writing 100%, revision 100%, S.Salmanzadeh; design 70%, initial writing 50%, search 50%.H.Abassifar; design 30%, writing 30%, search 50%.
